# Comment on Guerrero-Romero et al. Magnesium-to-Calcium Ratio and Mortality from COVID-19. *Nutrients* 2022, *14*, 1686

**DOI:** 10.3390/nu14163442

**Published:** 2022-08-22

**Authors:** Giuliana Scarpati, Ornella Piazza

**Affiliations:** Surgery and Dentistry “Scuola Medica Salernitana”, Department of Medicine, University of Salerno, 84084 Baronissi, SA, Italy

We read with great interest the article by Romero et al. “Magnesium-to-Calcium Ratio and Mortality from COVID-19” [1], which evaluated the association of the serum magnesium-to-calcium ratio with mortality in a cohort of 1064 COVID-19 patients. Magnesium supports the action of numerous anti-covid drugs [2] and the relevance of magnesium homeostasis for COVID-19 mortality has already been reported [3], while Romero et al., by comparing the data of 554 patients discharged per death with the data of 510 patients discharged per recovery, found that the best cut-off point for the magnesium-to-calcium ratio for identifying individuals at a high risk of mortality from COVID-19 was 0.20 (sensitivity 83%; specificity 24%). Nevertheless, ionized serum magnesium might be a better marker to identify critically ill patients with an impaired magnesium status, and accurately and routinely measuring ionized magnesium in critically ill patients may be helpful when replacing this micronutrient [4]. In our experience with 133 severe COVID-19 patients, who were admitted to ICU because of respiratory failure, we found that the Mg/Ca ratio was higher in the surviving patients than in the non-survivors (*p* < 0.001) and that a Mg/Ca ratio < 0.3 mg/dL was associated with a higher mortality (ROC curve Mg/Ca ratio: AUC = 0.565, J 0.24405, cut-off > 0.3; Sensitivity 0.28; Specificity 0.95). Moreover, we explored the association between the ionized-magnesium/ionized-calcium (iMg/iCa) ratio and mortality in the same population. The iMg/iCa ratio was higher in the surviving patients (Figure 1) and an iMg/iCa ratio of less than 0.55 mmol/L was associated with a higher mortality in patients with severe COVID-19. The analysis of the ROC curve for the iMg/iCa ratio (AUC 0.972; J 0.90909, cut-off 0.55) revealed a very high sensitivity (0.90) and specificity (1.00).

Interestingly, the serum magnesium and calcium values were within the normal range in all the 133 patients admitted to our COVID-19 ICU and no correlation between Mg and Ca serum levels and the well-known COVID-19-associated risk factors such as obesity, diabetes, or hypertension was found, nor was there a significant difference in the patients treated with invasive or non-invasive ventilation.

Although our outcomes are absolutely preliminary, if taken together with Romero’s results, they encourage Mg/Ca ratio use as a potential biomarker for phenotyping patients according to their disease severity, or as a prognosis biomarker. Nevertheless, even if a Mg/Ca ratio < 0.3 mg/dL and an iMg/iCa ratio < 0.55 are associated with a higher mortality, unfortunately, we do not possess enough knowledge to answer to the following question: does the supplementation of magnesium offer a therapeutical opportunity for severe COVID-19 patients?

## Figures and Tables

**Figure 1:**
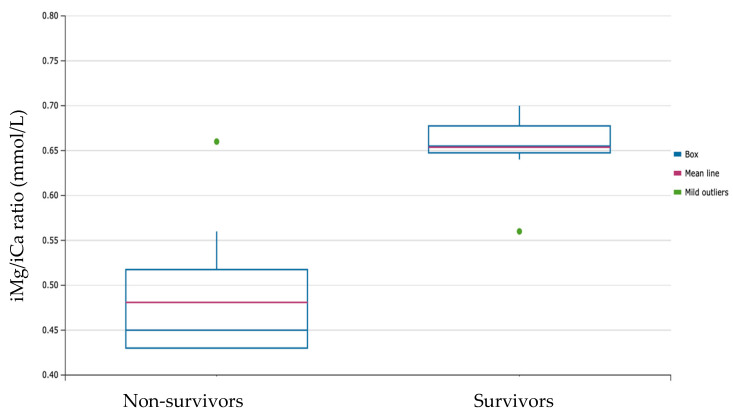
Box Plot iMg/iCa ratio by outcome.
